# Glitter-Like Iridescence within the Bacteroidetes Especially *Cellulophaga spp.*: Optical Properties and Correlation with Gliding Motility

**DOI:** 10.1371/journal.pone.0052900

**Published:** 2012-12-27

**Authors:** Betty Kientz, Adrien Ducret, Stephen Luke, Peter Vukusic, Tâm Mignot, Eric Rosenfeld

**Affiliations:** 1 UMR 7266 CNRS Littoral Environnement et Sociétés, University of La Rochelle, La Rochelle, France; 2 UMR 7283 CNRS Laboratoire de Chimie Bactérienne, Institut de Microbiologie de la Méditerranée, University of Aix-Marseille, Marseille, France; 3 School of Physics, University of Exeter, Exeter, United Kingdom; Loyola University Medical Center, United States of America

## Abstract

Iridescence results from structures that generate color. Iridescence of bacterial colonies has recently been described and illustrated. The glitter-like iridescence class, created especially for a few strains of *Cellulophaga lytica*, exhibits an intense iridescence under direct illumination. Such color appearance effects were previously associated with other bacteria from the *Bacteroidetes* phylum, but without clear elucidation and illustration. To this end, we compared various bacterial strains to which the iridescent trait was attributed. All *Cellulophaga* species and additional *Bacteroidetes* strains from marine and terrestrial environments were investigated. A selection of bacteria, mostly marine in origin, were found to be iridescent. Although a common pattern of reflected wavelengths was recorded for the species investigated, optical spectroscopy and physical measurements revealed a range of different glitter-like iridescence intensity and color profiles. Importantly, gliding motility was found to be a common feature of all iridescent colonies. Dynamic analyses of “glitter” formation at the edges of *C. lytica* colonies showed that iridescence was correlated with layer superposition. Both gliding motility, and unknown cell-to-cell communication processes, may be required for the establishment, in time and space, of the necessary periodic structures responsible for the iridescent appearance of *Bacteroidetes*.

## Introduction

Iridescence is a property of structural color arising from the interaction of light with micron- and sub-micron-sized periodic structures. The intense colors created exhibit angle-dependent color changes [1], [2], [3]. This form of color-appearance is found in natural or artificial organic/inorganic objects (*e.g.* opals, soap bubbles, compact discs) and in living organisms. Iridescence has been well studied in higher organisms, particularly Insecta [4], [5], [6], Aves [7], [8], [9], and fishes [2], [10]. Iridescence is also encountered in viruses [11], in marine organisms [12] and diatoms [13]. In animals, both communicative (*e.g.* species and sex recognition, predation avoidance) and non-communicative roles (*e.g.* photoprotection, thermoregulation, water repellency, friction reduction) were attributed to iridescence [14].

Iridescence expressed by bacterial colonies has been a vague concept in the literature. Only a few authors have studied the structural colors of prokaryotes [15], [16], [17], [18], [19], [20]. Ambiguous terms have been used for describing diverse visual effects of colonies or concentrated cell suspensions, but often with imprecise definitions. Images were rarely published and consequently the development of understanding of the visual colors was challenging. The comparison of a wide range of bacteria in [21] presented direct evidence of at least four categories of bacterial structural color. A novel and intense glitter-like iridescence class was created especially for a few strains of the marine bacterium *Cellulophaga lytica*. Glitter-like iridescence observed macroscopically under oblique direct illumination arose from bright millimeter- and sub-millimeter-sized centers of color distributed across the colony. The dominant glitter-like colors were observed to be green at the colonies' centers and were red and blue-violet at the colonies' peripheral edges. Importantly, and for the first time, the structural origin of the bacterial color appearance was verified by spectrophotometric data showing the reflected wavelengths under a broad range of light incidence angles. Several strains of *C. lytica* were compared and, interestingly, the intensity levels of iridescence were strongly different within the species.

The “iridescence” or “metallic tinge” of *C. lytica* colonies were mentioned only superficially in previous studies [22], [23]. Vague terms have been employed such as “greenish metallic iridescence” for the *Cellulophaga* genus [24] and “glistening”, “shine” or “shiny” for the *Bacteriodetes* [25]. These vague descriptions did not yield conclusions about the origin and nature of the observed colored appearances. Moreover, for some sample strains, relevant information relating to appearance was sometimes lost. For example, *Flavobacterium* [*Cytophaga*] *johnsoniae and “Sporocytophaga cauliformis”* were described as “greenish iridescent” [26] but without mention of the phenomenon in a more recent edition [24].

It was recently shown in *C. lytica* CECT 8139 that culture conditions can be the determining factor for the onset of iridescent appearance [27]. Thus, iridescent traits of many strains may have been overlooked due to non-optimal or inappropriate culture media conditions. In *Cellulophaga spp*. and other *Flavobacteriales* and *Cytophagales*, a close inspection of the optical properties may help to clarify the microbiology and ecology of iridescence.

In this study, we thoroughly examined the iridescence phenomena within all *Cellulophaga* species, and some closely related species from the *Bacteroidetes phylum*, on various culture media. Several *C. lytica* strains were included in order to evaluate the phenomenon within the species. *Bacteroidetes* strains described in previous studies as “green iridescent”, “iridescent”, “shiny” or “glistening” were selected to elucidate the color appearance effects. Illustrations of colonies are presented at the macroscopic and microscopic levels together with additional spectrophotometric data to describe the various intensities and iridescence profiles. Special attention was paid to the potential link between iridescence and gliding motility.

## Materials and Methods

### Bacterial strains

Strains are listed in Table 1 with recommended culture conditions and information on previous descriptions of their iridescence. “Reddish-greenish iridescence” and “metallic tinge” have been mentioned for Cellulophaga sp. [22], [26], so all strains belonging to this genus were included. Four *C. lytica* strains were incorporated in the study, including the genome-sequenced strain Lim 21^T^ also registered as DSM 7489 and CIP 103822 [28]. The non-iridescent mutant of *C. lytica* CECT 8139 was obtained by spontaneous mutagenesis after cell-suspension dilution and following culture growth on MA.

**Table 1:** Bacteria from *Bacteroidetes* phylum with culture conditions and previous mentions of iridescence.

Species	Strains	Origin	Country	Reference	Culture		Mention of iridescence	
					Medium	T°C	Description	Reference
**Bacteria with glitter-like iridescence**								
*Cellulophaga lytica*	CECT 8139	Surface of an anemone *Actinia equine*	Oléron island, France	[21]	MA	20°C	Glitter-like	[21]
	DSM 2040	Seawater aquarium	La Jolla, USA	[30]	CYT	20°C	Glitter-like	[21]
	DSM 7489^1(s)^	Beach mud from limon	Costa Rica				Reddish-greenish iridescence	[26] #
	CIP 103822^1(s)^						Greenish metallic iridescence	[24] #
	CIP 103822^1(s)^						Metallic tinge	[22]
**Bacteria with suspicion of glitter-like iridescence**								
*Promyxobacterium flavum*	DSM 3577	Rhizosphere of tomato plant	Russia	[43]	NA	25°C	Greenish iridescent	[26] #
*Flavobacterium [Cytophaga] succinicans*	DSM 4002	Eroded fin of a fingerling chinook salmon	USA	[26] #	CY	25°C	Greenish iridescent	[26] #
*Sporocytophaga cauliformis*	DSM 3657	Water	Lake Bodensee, Germany	[26] #	CY	20°C	Greenish iridescent	[26] #
*Flavobacterium [Cytophaga] johnsoniae*	DSM 2064(s)	ND	ND	[44]	CY	30°C	Almost greenish iridescent	[26] #
*Flavobacterium [Cytophaga] flevensis*	DSM 1076	Freshwater lake	The Netherlands	[32]	C/10	20°C	Iridescent	[26] #
**Bacteria with suspicion of iridescence**								
*Cellulophaga baltica*	CIP 106307	Surface of brown alga *Fucus serratus*	Baltic Sea, Denmark	[22]	MA	25°C	Metallic tinge	[22]
*Cellulophaga fucicola*	CIP 106308	Surface of brown alga *Fucus serratus*	Kattegat sea, Denmark	[22]	MA	25°C	Metallic tinge	[22]
*Cellulophaga pacifica*	LMG 21938	Seawater	Gulf Peter, Japan	[45]	MA	28°C	Shiny	[45]
*Psychroflexus [Flavobacterium] gondwanensis*	DSM 5423	Organic lake	Antarctica	[46]	MA	28°C	Shiny	[26] #
*Flavobacterium aquatile*	DSM 1132(s)	Deep well	Kent, England	[33]	Flavo	30°C	Glistening	[26] #
*Maribacter dokdonensis*	DSM 17201	Seawater	South Korea	[47]	MA	28°C	Glistening	[48]
**Bacteria with no description of iridescence**								
*Cellulophaga algicola*	DSM 14237(s)	Diatom *Melosira* and macrophyta	Eastern Antarctic coast	[49]	MA	10°C	-	-
*Cellulophaga tyrosinoxydans*	DSM 21164	Seawater	Korea	[50]	MA	28°C	-	-
*Cellulophaga geojensis*	CCUG 60801	Marine sand	Geoje island, Korea	[51]	MA	25°C	-	-
*Leeuwenhoekiella [Cytophaga] marinoflava*	DSM 3653	Seawater	Scotland	[26] #	CYT	20°C	-	-
*Flavobacterium degerlachei*	DSM 15718	Microbial mat	Antarctica	[52]	R2A	20°C	-	-

1 The strain DSM 7489 was also registered as Lim-21 and CIP 103822 [28].

(s) Genome sequenced strains.

# These references correspond to editions of the “Bergey's Manual of Systematic Bacteriology” or “The Prokaryotes”.

ND: not detailed.

### Culture conditions

Most of the culture media employed in this study comprised recommended media by strains catalogue DSMZ, CIP and BCCM/LMG (Table 1). For each strain, the culture media tested and the growth temperatures are listed in Table 2. Bacteria from the *Bacteroidetes* phylum have marine, freshwater or soil origins, thus both marine media (i) and terrestrial media (ii) were prepared.

**Table 2:** Iridescence phenomenon of strains belonging to the *Bacteroidetes* phylum.

Strains		Media										T°C
		MA		SYP		CYT		LN		sNA		
		*Growth* 1	*Iridescence* 2	*Growth*	*Iridescence*	*Growth*	*Iridescence*	*Growth*	*Iridescence*	*Growth*	*Iridescence*	
**Marine strains**												
*Maribacter dokdonensis* **	DSM 17201	++	+ G	++	+ G	++	+++ G	++	+ G	+	−	28°C
*Cellulophaga lytica* *	CECT 8139	++	+++ G/R	++	++ G/R	++	++ G/R	++	++ G	+	−	25°C
	DSM 2040	++	+++ G/R	++	++ G/R	++	+++ G/R	++	+++ G	+	−	25°C
	DSM 7489(s)	++	+ G/R	++	+ G/R	++	+ G/R	++	+ G	+	−	25°C
	DSM 2039	++	−	++	−	++	++ Y	++	++ G	+	−	25°C
	CIP 103822	++	−	++	−	++	−	++	−	+	−	25°C
*Cellulophaga baltica* *	CIP 106307	++	+ R	++	+ R	++	+++ R	++	+ R	+	−	25°C
*Cellulophaga fucicola* *	CIP 106308	++	+ G/R	++	+ G/R	++	++ G/R	++	+ G	+	−	25°C
*Cellulophaga pacifica* **	LMG 21938	++	+ G/R	++	+ G/R	++	++ G/R	++	−	+	−	25°C
*Cellulophaga geojensis*	CCUG 60801	++	+ G/R	++	+ G/R	++	++ G/R	++	++ G	+	−	25°C
*Cellulophaga tyrosinoxydans*	DSM 21164	++	−	++	−	++	++ G	++	+ G	+	−	25°C
*Cellulophaga algicola*	DSM 14237(s)	+	−	++	−	++	−	+	−	−		25°C
*Psychroflexus gondwanesis* **	DSM 5423	++	−	++	−	+	−	+	−	+	−	25°C
*Zobellia uliginosa*	DSM 2061	++	−	++	−	+	−	+	−	+	−	25°C
*Leeuwenhoekiella marinoflava*	DSM 3653	++	−	++	−	+	−	+	−	+	−	20°C
*Flavobacterium degerlachei*	DSM 15718	++	−	++	−	+	−	+	−	+	−	20°C
Terrestrial strains		CY		Flavo		C/10		TSA		NA		
*Flavobacterium johnsoniae* *	DSM 2064(s)	++	++ G/R	++	+ G/R	++	++ G/R	++	−	++	+ G/R	30°C
*Flavobacterium aquatile* **	DSM 1132(s)	++	−	++	−	++	−	++	−	++	−	30°C
*Flavobacterium succinicans* *	DSM 4002	++	+ R	++	−	++	−	++	−	++	−	25°C
*Promyxobacterium flavum* *	DSM 3577	++	−	++	−	++	−	++	−	++	−	25°C
*Flavobacterium flevensis* *	DSM 1076	++	−	++	−	++	−	++	−	++	−	20°C
*Sporocytophaga cauliformis* *	DSM 3657	++	+ R	++	−	++	+ R	++	−	++	+ R	20°C

* Strains previously described with “metallic tinge” or “greenish” appearances.

** Strains previously described with “glistening” or “shiny” appearances.

1 Apparent growth: −, none; +, moderate; ++, good; +++, intense.

2 Iridescence and observed colors: −, none; +, low; ++, good; +++, bright. R, red; Y, Yellow; G, Green; G/R, middle green with red edges.

(s) Genome sequenced strains.

**(i) Marine media** comprised ready-to-use Marine agar (MA) medium from Dutscher (Laboratorios Conda, S.A. Pronadisa®) and salted Nutrient agar (sNA) (Dutscher) with a supplement of NaCl 25 g L^−1^. Prepared media were made with artificial seawater (ASW) Instant Ocean^©^ (30 g L^−1^). Cytophaga agar (CYT) contained 1 g tryptone, 0.5 g yeast extract, 0.5 g CaCl_2_.2 H_2_O, 0.5 g MgSO_4_.7 H_2_O, and 15 g agar in 1 L of ASW [22], [29], [30]. This medium was adapted with casein replaced by tryptone because *C. lytica* does not degrade casein. Low nutrient medium (LN) contained 15 g of agar in 1 L of ASW [31]. Recommended for *Zobellia uliginosa*, Seawater yeast peptone agar (SY) contained 5 g peptone, 3 g yeast extract, 12 g agar, 250 ml distilled water and 750 ml ASW.

**(ii) Terrestrial media** comprised ready-to-use Trypticase soy agar (TSA) and Nutrient agar (NA) (Dutsher). CY-agar (CY) was prepared by adding 3 g casitone, 1.36 g CaCl_2_.2H_2_O, 1 g yeast extract and 15 g of agar in 1 L of distilled water [26]. Recommended for *Flavobacterium flevensis*, C/10 contained 3 g casitone, 1.36 g CaCl_2_.2H_2_O and 15 g of agar in 1 L of distilled water [32]. *Flavobacterium aquatile* medium (Flav) was prepared by adding 2 g Na-caseinate, 0.5 g yeast extract, 1 g proteose peptone, 0.5 g K_2_HPO_4_ and 15 g of agar in 1 L of distilled water [33], [34].

### Imaging colonies' iridescence appearance

The iridescence appearance of colonies was observed with the aid of a streaking procedure [21]. One colony from a 24 h-old plate was subcultured on a new plate drawing a 5 cm-linear streak in triplicate. Cultures were photographed after 24 h of growth in a dark room using an experimental arrangement of oblique epi-illumination at a fixed illumination angle of 60°. The light source was a lamp (Kaiser RB 218 N HF copy lighting unit) of 18 W, 5400 K, the operating voltage correspond to AC 220–240 V, 50 Hz with an operating frequency of 40 kHz. The camera was a Nikon D1500 18–55 VR on Av program with f 22 (12.1 Mega pixels). The lens was a macro, large-size used in superfin mode.

### Gliding motility

Gliding motility was observed using timelapse microscopy. Pictures of colony edges were taken using an Olympus SZ61 stereomicroscope every 1 min for 20 min. The plates were observed under transverse illumination using a SCOTT KL 1500-halogen light source (150 W). For closest observations, colony edges were also observed in phase-contrast using an automated and inverted epi-fluorescence microscope TE2000-E-PFS (Nikon, France). The microscope was equipped with “The Perfect Focus System” that automatically maintains focus on a point of interest. Images were recorded with a CoolSNAP HQ 2 (Roper Scientific, Roper Scientific SARL, France) and a 40×/0.75 DLL “Plan-Apochromat” or a 100×/1.4 DLL objective. Due to the short working distances of 100× or 40× objectives, a 1 cm sided cube of colony growing on agar was cut and inverted on a coverslip.

### Physical measurement of glitter-like iridescence (microspectrophotometry)

The diverse intensity and iridescence profiles of glitter-like iridescence were measured by microspectrophotometry [35]. Selected bacterial strains were included so that their various color-appearance effects could be evaluated. Samples were epi-illuminated and reflected light was collected from the same side of the sample. The angle of illumination and of detection could be separately set and controlled to a resolution of 0.5°. The illumination plane and the detection plane were offset from each other by 3° to enable unobstructed detection over the full collection angle range. Illumination was directed onto the sample through an Ocean Optics UV-Vis-NIR optical fiber that was connected to an Ocean Optics HPX-2000 light source, spanning approximately 300 nm to 850 nm. The reflected light was collected using a similar optical fiber that was connected to an Ocean Optics USB 4000-UV-VIS spectrometer. For a series of chosen fixed-incidence illumination angles, the collection fiber was stepped in 5° angle steps in an arc over the sample, and reflection spectra were recorded at each position. In this way, the dependence of reflected color with angle, and hence the extent of each sample's iridescence, could be measured and assessed.

## Results

### Iridescence within the Bacteroidetes phylum

**Figure 1:**
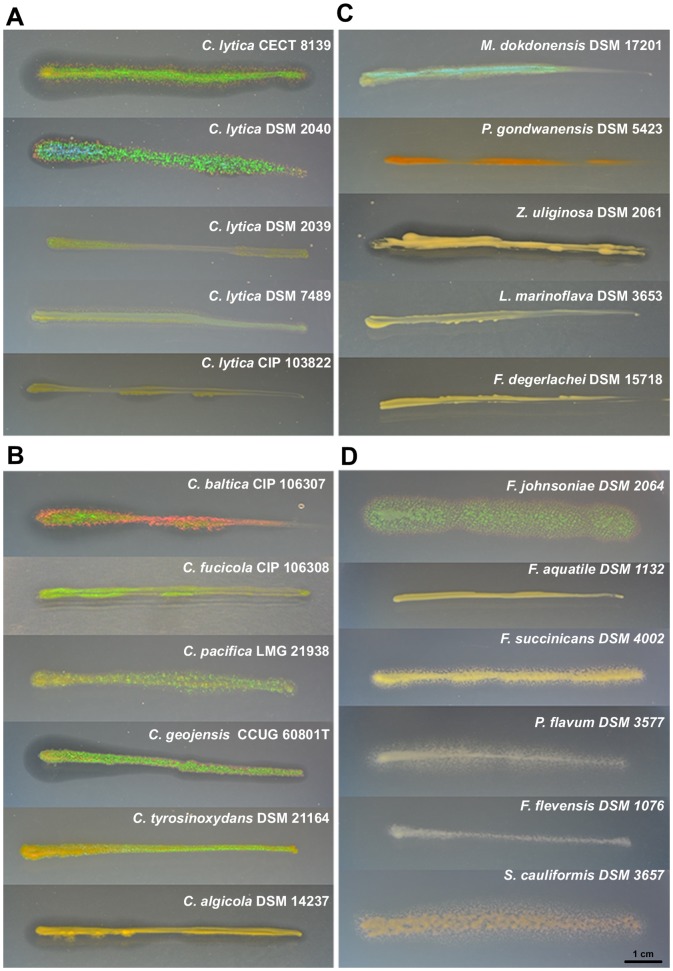
Iridescence colors of *Bacteroidetes*. Iridescence was observed after 24 h of growth (or 48 h for *Z. uliginosa* and *P. gondwanensis*) on CYT medium (A, B, C), for marine strains, or CY medium (D) for terrestrial strains.

In relation to previous descriptions of optical effects (Table 1), the glitter-like iridescence of several *Bacteroidetes* strains was investigated on several culture media (Table 2). The best media for iridescence observation were CYT and CY respectively for marine and terrestrial strains. The sNA and TSA-rich media did not appear to give rise to discernable iridescence. The brightness of the iridescence and the observed colors previously reported as having an “iridescent profile” were different within the species (Table 2, Figure 1). However, the glitter-like iridescence that was defined as being green with red edges was common to most iridescent *Bacteroidetes*. Red or yellow iridescence was also observed in a few species. A green iridescence profile was typical of most transparent colonies growing on LN medium. *C. baltica* exhibited a red iridescence after 24 h of growth but could acquire the green and red iridescence after 48 h/72 h (data not shown). Except for *C. algicola* and *C. lytica* CIP 103822, all *Cellulophaga spp*. were iridescent (Figure 1 A, B).

Three terrestrial strains were iridescent. *F. johnsoniae* had moderate iridescence intensity which was higher than the other strains, *S. cauliformis* and *F. succinicans* (Figure 1 D).

### Spectrophotometric profiles of glitter-like iridescences

**Figure 2:**
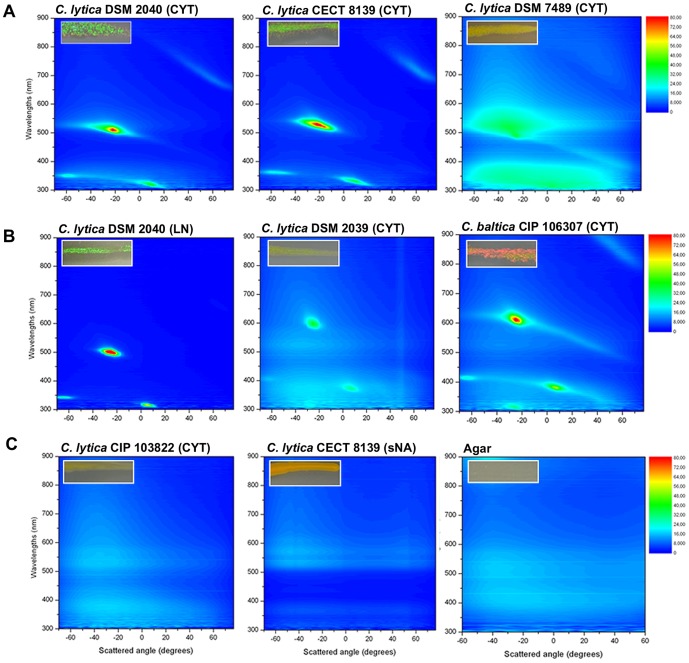
Spectral properties of the different glitter-like iridescence profiles. The color maps show the angle-dependent spectral reflectance of *Cellulophaga spp*. colonies grown 24 h on different media. Samples were illuminated at a fixed light angle of −70°. Scattered wavelengths from 300 nm to 850 nm were recorded at different detection angles from −65° to 70° with 5° angle step resolution. Results are shown using a color intensity scale. As shown in the small inserts (upper left in each color map), iridescence profiles correspond to: (A) green and red iridescence profile with high intensity from *C. lytica* DSM 2040, moderate intensity from *C. lytica* CECT 8139, and low intensity from *C. lytica* DSM 7489; (B) other color profiles with green profile from *C. lytica* DSM 2040 on LN, yellow profile from *C. lytica* DSM 2039 and red iridescence profile from *C. baltica* CIP 106307; (C) non-iridescent controls with *C. lytica* CIP 103822, *C. lytica* CECT 8139 on sNA and the CYT agar control. Wavelength values: UV, <400 nm; violet, 400 to 435 nm; blue, 435 to 490 nm; cyan, 490 to 520 nm; green, 520 to 560 nm; yellow, 560 to 590 nm; orange, 590 to 620 nm; red, 620 to 700 nm; and infrared, >700 nm.

Optical reflection associated with different glitter-like iridescent profiles observed macroscopically was quantified using microspectrophotometry (Figure 2). The data shows optical reflection bands that unequivocally represent iridescence, and therefore structurally-generated color, of bacterial colonies on account of the change in their reflected wavelengths with angle. A common form of spectral map (presenting sample intensity *vs* wavelength *vs* detection angle) was typical to the iridescence profiles of all structurally colored samples. Main reflected wavelengths (700 nm, 530 nm, 380 nm, and 310 nm) are represented by the areas with higher intensity in coloring, visible for example on *C. lytica* DSM 2040 (Figure 2 A). A higher intensity of green-red (G/R) iridescence results in narrower band of reflected wavelengths (Figure 2 A).

The main scattering peaks were conserved for the different color profiles but with a displacement within the spectra (Figure 2 B). For the green profile of LN-grown colonies, the main peak was at lower wavelengths, *i.e* at 500 nm instead of 530 nm for the G/R profile (Figure 2 B). Higher wavelengths at 600 nm and 635 nm were obtained for yellow and red iridescent profiles, respectively. A fifth peak at 380 nm was discerned for the red iridescent *C. baltica* (Figure 2 B). Under non-iridescent conditions with *C. lytica* CIP 103822 on CYT medium and *C. lytica* CECT 8139 on sNA, no reflection peaks were observed. The background of reflected colors was due to pigmentation and the agar medium (Figure 2 C).

### Correlation between gliding motility and iridescence

**Figure 3:**
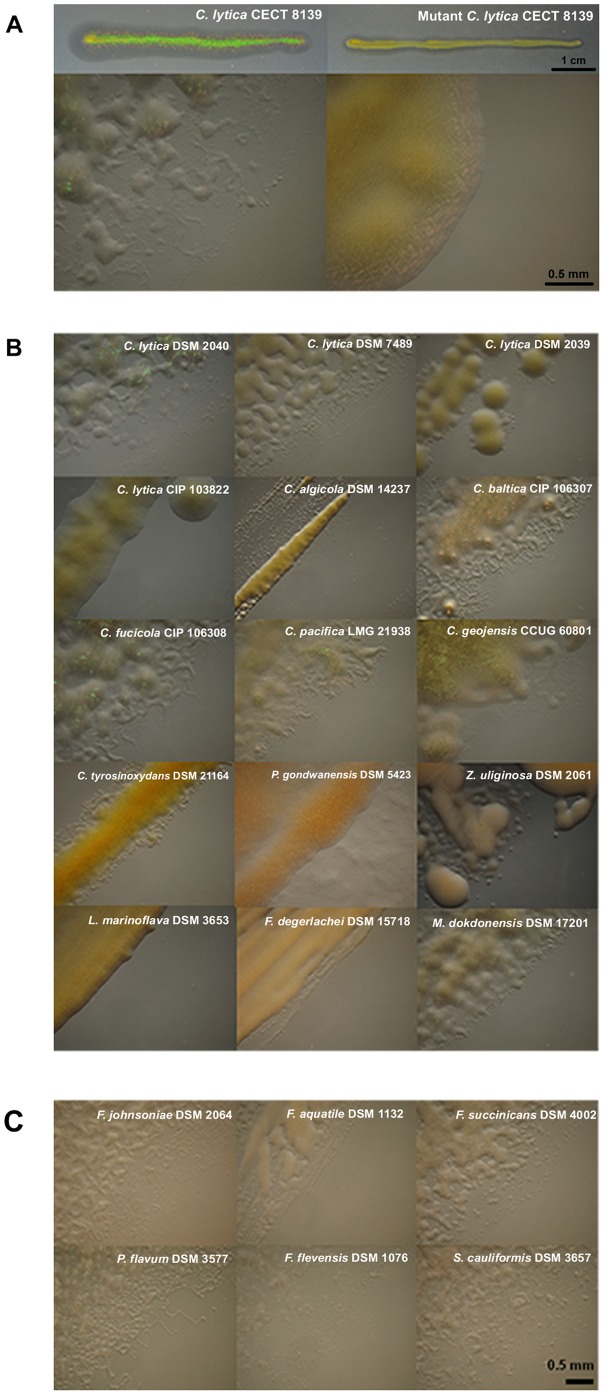
Edges patterns of *Bacteroidetes* colonies. Marine bacteria (A, B) and terrestrial bacteria (C) were grown 24 h on CYT and CY media, respectively. In (A), pictures of the entire colonies of *C. lytica* CECT 8139 and its mutant (impaired in gliding motility) are shown for comparison at the macroscopic level. The mutant was not iridescent on SYP, CYT, LN and sNA culture media (data not shown). All iridescent colonies exhibited typical spreading and jagged edges. Iridescent strains were: *C. lytica* CECT 8139 and DSM 2040 (and to a lesser extent DSM 7489), *C. baltica* CIP 106307, *C. fucicola* CIP 106308, *C. pacifica* LMG 21938, *C. geojensis* CCUG 60801, *C. tyrosinoxydans* DSM 21164, *M. dokdonensis* DSM 1720, *F. johnsoniae* DSM 2064, *F. succinicans* DSM 4002 and *S. cauliformis* DSM 3657.

Gliding bacteria move individually or in swarms, resulting in the formation of typical colonies that have thin spreading edges [36]. Gliding motility might be required for the establishment of periodic structures within the iridescent colonies. In order to test this hypothesis, we attempted to isolate a mutant of *C. lytica* CECT 8139 impaired in gliding motility. A spontaneous gliding-negative mutant was obtained. Interestingly, this mutant is almost non-iridescent, harboring only a residual iridescence when the colony edges were observed microscopically (Figure 3 A). A strong link between gliding motility and wild-type iridescence may thus exist. To reinforce this hypothesis, a rigorous microscopic inspection of colony edges was performed in the *Bacteroidetes* strains (Figure 3 B, C). Since iridescence and colony edges were not in the same focal plane, the optical effects were hard to observe in this test. All iridescent colonies exhibited spreading edges, a hallmark of gliding bacteria. Interestingly, these typical edges were absent in colonies that appeared non-iridescent at the macroscopic level (*e.g. C. algicola* and *C. lytica* CIP 103822). However, typical edges of gliding bacteria were found in some non-iridescent strains, in particular for terrestrial bacteria. Moreover, no correlation was found between the intensity of iridescence and the gliding rates for the tested strains (Table 3).

**Table 3:** Gliding motility and iridescence on CYT or CY media.

Strains		Iridescence^1^	Gliding motility	
Marine strains (CYT medium)			Typical edges^2^	Rate^3^
*Cellulophaga lytica*	CECT 8139	++	++	Fast
	CECT 8139 mutant	∼	−	
	DSM 2040	+++	++	Fast
	DSM 7489(s)	+	+	Moderate
	DSM 2039	++	+	Slow
	CIP 103822	−	−	
*Cellulophaga baltica*	CIP 106307	++	++	Slow
*Cellulophaga fucicola*	CIP 106308	++	++	Moderate
*Cellulophaga pacifica*	LMG 21938	++	++	Moderate
*Cellulophaga geojensis*	CCUG 60801	++	++	Slow
*Cellulophaga tyrosinoxydans*	DSM 21164	++	+	Slow
*Cellulophaga algicola*	DSM 14237(s)	−	−	
*Psychroflexus gondwanensis*	DSM 5423	−	−	
*Zobellia uliginosa*	DSM 2061	−	+	Moderate
*Leeuwenhoekiella marinoflava*	DSM 3653	−	−	
*Flavobacterium degerlachei*	DSM 15718	−	−	
*Maribacter dokdonensis*	DSM 17201	++	++	Slow
**Terrestrial strains** (CY medium)				
*Flavobacterium johnsoniae*	DSM 2064(s)	++	++	Moderate
*Flavobacterium aquatile*	DSM 1132(s)	−	−	
*Flavobacterium succinicans*	DSM 4002	+	++	Slow
*Promyxobacterium flavum*	DSM 3577	−	++	Moderate
*Flavobacterium flevensis*	DSM 1076	−	++	Slow
*Sporocytophaga cauliformis*	DSM 3657	+	++	Fast

1 Iridescence intensity: −, none; ∼, microscopically observed; +, low; ++, good; +++, intense.

2 Typical gliding edges: −, none; +, moderate; ++, wide.

3 Gliding motility rates were qualified visually: Fast, spreading movements on the agar with visible distance covered after only 20 min; Moderate, spreading movements at a lower extend; Slow, few movements within the colony with short distance covered.

(s) Genome sequenced strains.

**Figure 4:**
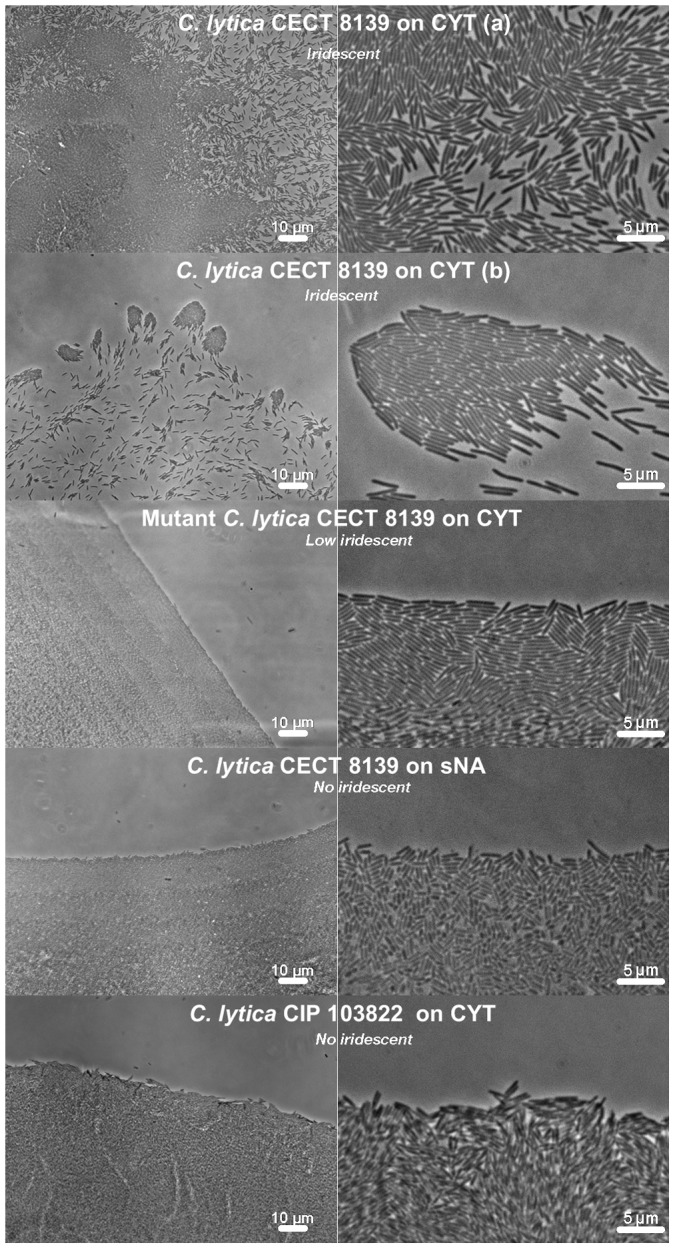
Phase contrast observation of colony edges of different *C. lytica* strains at high magnifications. Iridescent *C. lytica* CECT 8139 growing on CYT was observed at different colony positions: an edge (a) and an extreme edge (b). Low or non-iridescent conditions were CECT 8139 gliding defective mutant on CYT, *C. lytica* CECT 8139 grown on sNA and CIP 103822 strains grown on CYT. Magnification was ×400 (left column) and ×1000 (right column).

Colony edges of *C. lytica* strains were also observed at higher magnification (Figure 4). *C. lytica* CECT 8139 moved as single cells and within coordinated groups. The mutant of *C. lytica* CECT 8139 displaying only a reduced iridescence at the colony edges still formed tiers but lacked gliding cells. Moreover, when *C. lytica* CIP 103822 and *C. lytica* CECT 8139 were plated in non iridescent conditions (CYT and sNA respectively), gliding cells were not observed. Conversely, iridescent *C. lytica* CECT 8139 exhibited fast gliding motility (see Movies S1 and S2 as supplemental data). Taken together these results suggest a correlation between iridescence and gliding motility.

### Dynamic formation of an iridescent glitter

**Figure 5:**
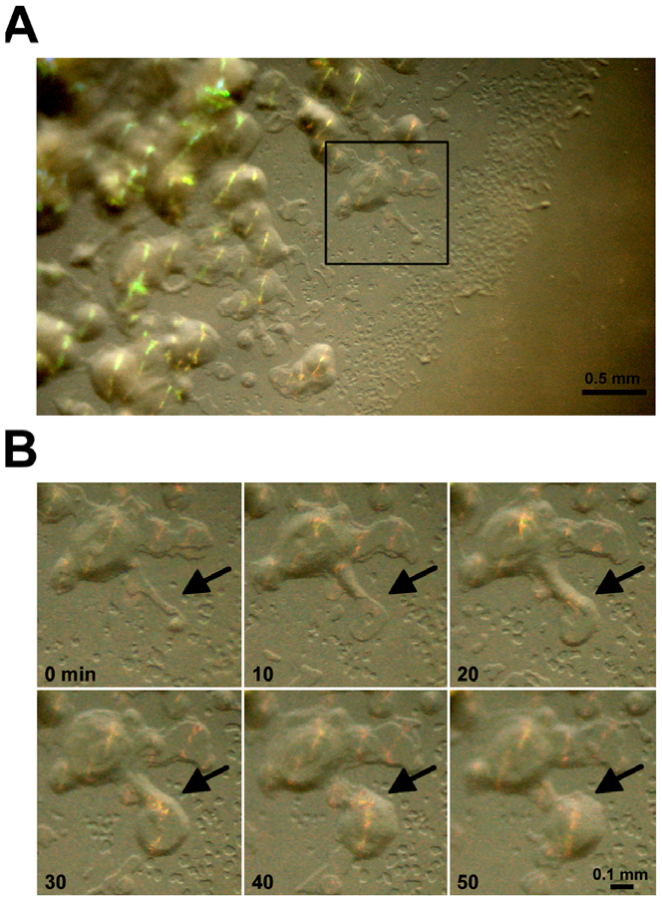
Dynamic formation of iridescence. A glitter centre (black arrow) was observed on the edges of a *C. lytica* DSM 2040 colony after 24 h incubation on CYT medium. Colony was photographed every 5 min for 60 min. Scales bars (A) 0.5 mm and (B) 0.1 mm.

We designed an experimental device to monitor the appearance of iridescence over time in a gliding colony. Intense iridescent colonies of *C. lytica* DSM 2040 were used to observe the kinetic formation of glitters (Figure 5). Colonies appeared to move rapidly and the formation of a glitter centre could be followed (black arrows). After only 20 minutes the first non-iridescent layer was visualized and 10 minutes later, likely due to layer superposition, a glitter effect could be observed. The iridescence was conserved even with rapid micro-colony movement, suggesting the existence of a complex ordered structure continuously being formed, for example, if the cells follow the exact same path in the glitter area. These effects were more easily observed on the video sequence (see Movie S3).

## Discussion

Iridescence in prokaryotes is a concept that has only recently been elucidated [21]. The intense glitter-like iridescence observed under direct illumination was typical of some *C. lytica* colonies. Understanding the bases of such iridescence and its cellular function requires its identification in bacteria and its correlation with the environment and specificities of the iridescent strains. Therefore, we set out to document iridescence in the *Bacteroidetes* phylum. We found that the previous descriptive terms such as “shiny”, “glistening”, “metallic tinge” or “greenish iridescent” were clearly correlated with iridescence effects for marine bacteria, but this correlation was not clear for terrestrial strains. Marine bacteria and in particular *Cellulophaga spp*. were found to exhibit the most intense iridescence effects. However, the iridescent trait was scattered within the *Bacteroidetes* and no phylogenetic link was found between the iridescent species.

As recently shown by [27], culture conditions were confirmed to influence strongly the observation of the iridescence. Nutrient-rich media were found to inhibit both gliding motility and iridescence. Specific physiological states might thus be required for the production of metabolites involved in the onset of iridescence. The repression of iridescence under certain conditions may explain, in part, the lack of previously reported descriptions and illustrations.

As several strains did not produce the previously reported effects it was essential to illustrate colonies' iridescence properties precisely. This was achieved using a specific epi-illumination experimental arrangement. Descriptive terms must be chosen carefully and the observation conditions must be precisely defined, in particular because a diverse set of iridescence profiles have been highlighted in this study. The green and red iridescence defined as “glitter-like” iridescence was observed in several marine *Bacteroidetes* and *F. johnsoniae* but with different intensity levels. Evidence of distinct color variations was also collected. For these reasons, the common glitter-like iridescence class could be divided in different sub-classes referring to the color.

In spite of these differences in intensities and/or reflected colors, strong similarities were found between the intensity-color maps of their iridescence. Similar periodic structures, with minor variations of dimensions and periodicity, are therefore inferred to be responsible for the iridescent appearances observed.

It is still unknown whether bacterial iridescence occurs in natural habitats. However, iridescence was conserved under conditions that mimic the marine habitat [27]. Moreover, *Bacteroidetes* are widely distributed in nature, and *Cellulophaga spp*. are found in rocky shore ecosystems where stressful conditions of salinity, temperature variation, desiccation and light exposure select adapted organisms. The iridescence of bacterial colonies or biofilms might therefore have eco-biological roles by strongly reflecting specific wavelengths. UV and IR wavelengths were particularly well reflected by colonies exhibiting glitter-like iridescence. These typical reflections may have photoprotective or thermoregulatory roles. Non-communicative functions of iridescence have been reported in higher organisms [2], [6], [14]. For example, the reed frog *Hyperolius viridiflacus* adapts its skin color appearance and reflectance with the arid environmental conditions of African savannas [37]. The measured spectral maxima in reflectance are in the IR wavelengths from 800 nm to 1100 nm with additional reflectance of UV. Periodic structures are also responsible for reducing mechanical friction in some burrowing animals [38]. A similar mechanical role might also be envisaged for bacterial iridescence.

A strong correlation was found between gliding motility and iridescence in this study. Gliding motility appeared as a necessary -but not sufficient- condition for the formation of iridescent colonies. In gliding motility, the cell movements proceed without pili or flagella, but correspond to “a smooth translocation of cells over a surface by an active process that requires the expenditure of energy” [36]. The cellular mechanisms are not perfectly known in the *Bacteroidetes* [39]. At the colony level, one of the characteristics is spreading edges. In this study, all iridescent colonies possessed typical edges of gliding bacteria, and the motility was particularly rapid for some strains. Moreover, dynamic analyses of glitter-centre formation indicated that the iridescent structures themselves could move. A fast gliding motility was thus compatible with the maintenance of a certain periodicity of disposition within the cell population. Such process likely requires fine-tuning and a high degree of cell-to-cell communication. This is a major question that remains unanswered. Among the strains tested, only three have their genome completely sequenced, namely *F. johnsoniae* DSM2064 (UW101), *C. algicola* DSM 14237 and *C. lytica* DSM 7489 [28], [40]. *F. johnsoniae* might constitute a good candidate to characterize the genetic bases of iridescence. Accordingly, *F. johnsoniae* has already been used as a model organism for studies of *Cytophaga-Flavobacterium* gliding motility, and a wide variety of genetic tools have been developed for its manipulation [41], [42]. Moreover, genome comparisons between iridescent and non-iridescent *Bacteroidetes* strains might help us to identify key genes controlling both gliding motility and dynamic formation of iridescent glitter centers. This approach is currently being used in our laboratory for several strains of *C. lytica*.

## Supporting Information

Movie S1 **Gliding motility of** ***C. lytica*** **CECT 8139 observed at colony edges by phase contrast microscopy.** The bacterium was grown on Cytophaga agar medium and movements were recorded during 1 minute (one photo every three seconds). Gliding reverse movements were visible. Motility was reduced in the central parts of the colony.
(MOV)

Movie S2 **Gliding motility of** ***C. lytica*** **CECT 8139 observed at colony extreme edges by phase contrast microscopy.** The bacterium was grown on Cytophaga agar medium and movements were recorded during 1 minute (one photo every three seconds). A collective motion and the organization of cells into larger moving clusters were observed.
(MOV)

Movie S3 **Dynamic formation of an iridescent glitter on the edges of a** ***C. lytica*** **DSM 2040 colony after 24 h incubation on Cytophaga agar medium.** The phenomenon was observed with a total duration of 50 minutes (one photo every minute). On the edges, spreading movements were well visible. Several layers seemed needed to observe the red iridescence from the glitter.
(MOV)
